# A Novel Neurofibromatosis Type 1 (NF1) Frameshift Mutation Associated With Atypical Hypotensive Pheochromocytoma: A Case Report

**DOI:** 10.7759/cureus.108246

**Published:** 2026-05-04

**Authors:** Rojda Kotan, Ensar Gazi Göçer, Mehmet Sait Koç

**Affiliations:** 1 Endocrinology and Metabolism, Inonu University Faculty of Medicine, Malatya, TUR

**Keywords:** adrenal pheochromocytoma, case report, hypotension, metanephrine, neurofibromatosis type 1 (nf1)

## Abstract

Neurofibromatosis type 1 (NF1) is an autosomal dominant tumor predisposition syndrome associated with an increased risk of pheochromocytoma. Pheochromocytoma typically presents with hypertension and catecholamine excess; however, atypical clinical features may occur. A 58-year-old Turkish male patient with clinically diagnosed NF1 was evaluated for persistent hypotension (approximately 90/50 mmHg) without classic catecholamine-related symptoms. Biochemical testing showed isolated elevation of plasma and urinary metanephrine, while normetanephrine and other catecholamines were within reference ranges. Imaging revealed a 3.7 cm left adrenal mass with increased uptake on Gallium-68 DOTA-octreotate Positron Emission Tomography/Computed Tomography (Ga-68 DOTATATE PET/CT). The patient underwent left adrenalectomy after carefully titrated preoperative alpha-blockade due to baseline hypotension. Histopathological findings were consistent with pheochromocytoma. Genetic analysis using next-generation sequencing identified a heterozygous NF1 frameshift variant (c.3322dup p.(Thr1108AsnfsTer9)), predicted to result in a premature termination codon and classified as pathogenic according to American College of Medical Genetics and Genomics (ACMG) / Association for Molecular Pathology (AMP) criteria. Postoperatively, metanephrine levels normalized and blood pressure remained stable. Pheochromocytoma in patients with NF1 may present with hypotension and isolated biochemical abnormalities. This possibility should be considered in the appropriate clinical context. The identified NF1 variant contributes to the existing literature on NF1-associated tumors.

## Introduction

Neurofibromatosis type 1 (NF1) is a common autosomal dominant neurocutaneous disorder with an estimated prevalence of approximately one in 3,000 individuals [1,2]. It is caused by pathogenic variants in the NF1 gene located on chromosome 17q11.2, encoding neurofibromin, a tumor suppressor protein involved in Ras signaling regulation [3,4]. Loss-of-function variants result in increased cellular proliferation and tumor predisposition [5].

Pheochromocytoma is a recognized but relatively uncommon endocrine manifestation of NF1, with a reported prevalence ranging from 0.1% to 5.7% depending on screening strategies [6,7]. Classically, pheochromocytoma presents with paroxysmal or sustained hypertension accompanied by headache, palpitations, and diaphoresis. Current guidelines emphasize plasma free metanephrines as the preferred biochemical screening test [8].

However, emerging evidence suggests that pheochromocytoma may present atypically, including normotension or even hypotension, particularly in tumors with selective catecholamine secretion patterns or altered adrenergic receptor responsiveness [9-11]. Such presentations may delay diagnosis, especially in patients lacking classic symptomatology.

Although pheochromocytoma classically presents with hypertension, atypical presentations such as normotension or hypotension have been increasingly recognized. These unusual presentations may lead to diagnostic delay, particularly in patients without classical catecholamine-related symptoms. Reported rates of normotensive presentations range from approximately 10% to 15%, while hypotensive cases remain rare. Despite this emerging awareness, there is limited data regarding atypical hemodynamic presentations of pheochromocytoma in patients with NF1. This creates a diagnostic challenge and highlights the importance of maintaining clinical suspicion even in the absence of hypertension [6,7].

Here, we report a patient with NF1 presenting with persistent hypotension and isolated metanephrine elevation, representing a rare and diagnostically challenging manifestation. In addition, we describe a novel pathogenic NF1 frameshift mutation, further expanding the clinical and genetic spectrum of NF1-associated pheochromocytoma [12-16].

## Case presentation

Patient information

A 58-year-old man of Turkish origin was referred to the endocrinology clinic for evaluation of poorly controlled type 2 diabetes mellitus. His identity was anonymized in accordance with ethical standards. The patient did not report episodic headache, palpitations, diaphoresis, anxiety attacks, or other paroxysmal symptoms suggestive of catecholamine excess. Additionally, there was no history of hypertensive episodes. The patient had a 10-year history of type 2 diabetes mellitus. He had no known family history of pheochromocytoma or other endocrine tumors. There was no relevant psychosocial stressor or history of substance use. Genetic evaluation was pursued due to clinical features consistent with NF1. The patient reported no known family history of NF1 or related clinical manifestations. Furthermore, no first-degree relatives were known to have café-au-lait macules, neurofibromas, or endocrine tumors. Prior to referral, the patient had received standard medical therapy for diabetes mellitus. There was no history of previous surgical interventions related to adrenal or endocrine disease.

Clinical findings

**Figure 1:**
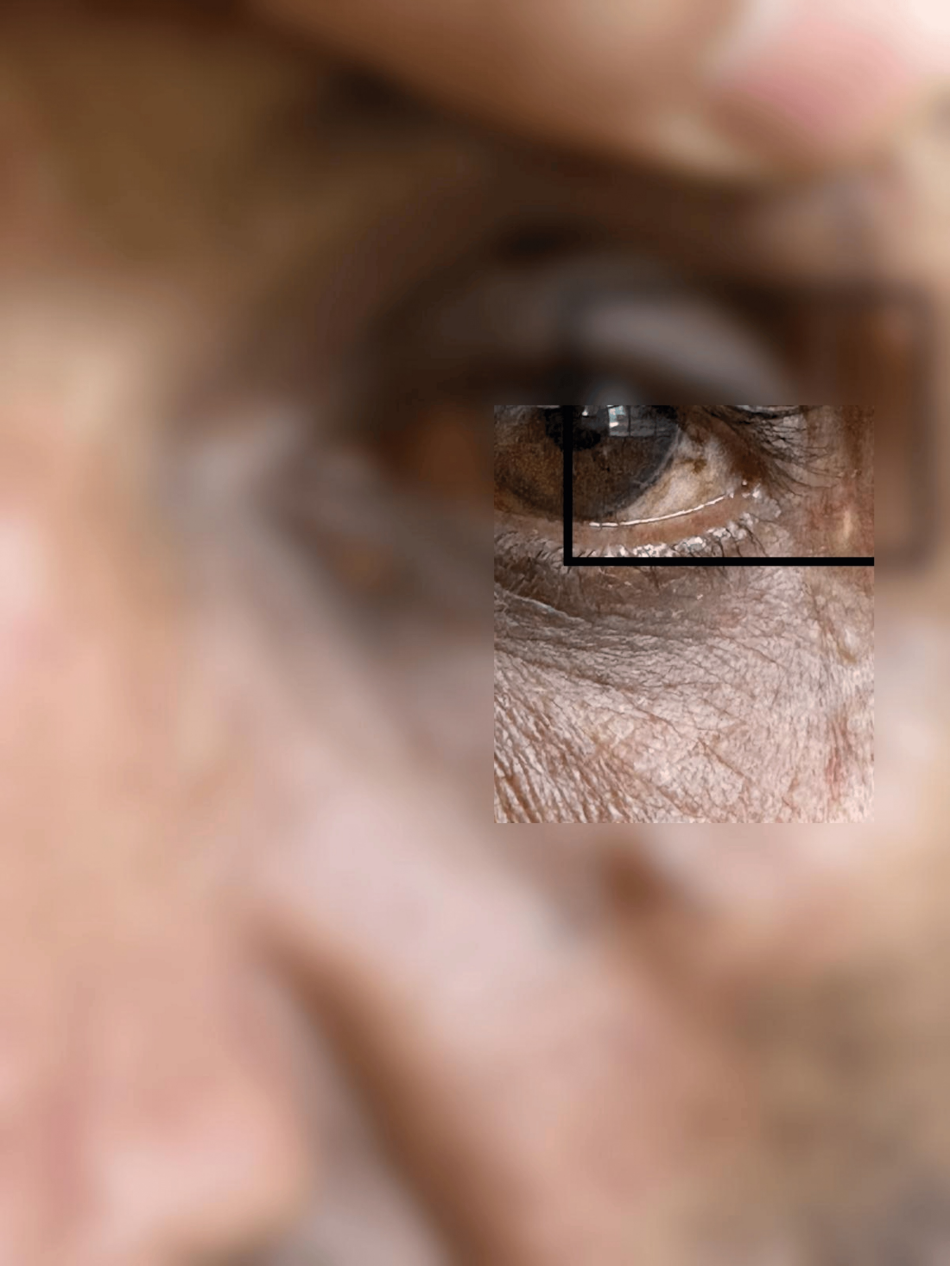
Lisch nodules of the iris, a characteristic ophthalmologic feature supporting the diagnosis of NF1

**Figure 2:**
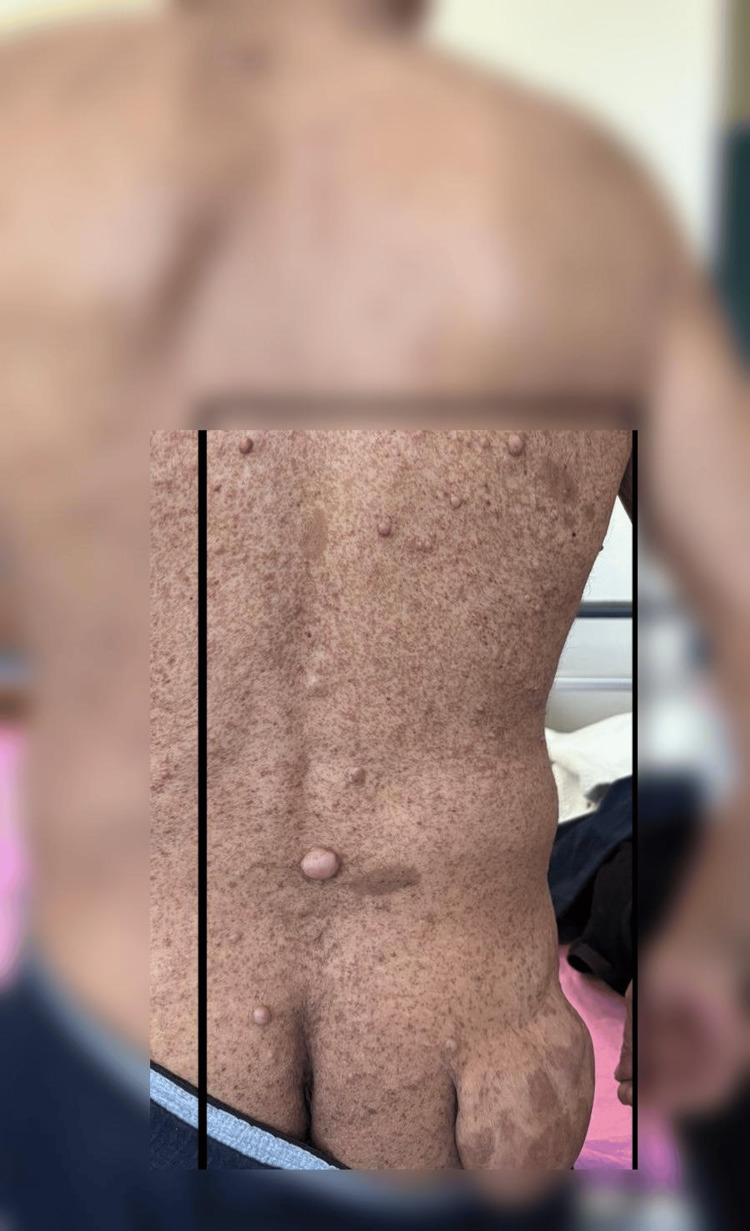
Giant plexiform neurofibroma, a hallmark manifestation of NF1 with potential for significant morbidity and café-au-lait macules, typical dermatologic findings

**Figure 3:**
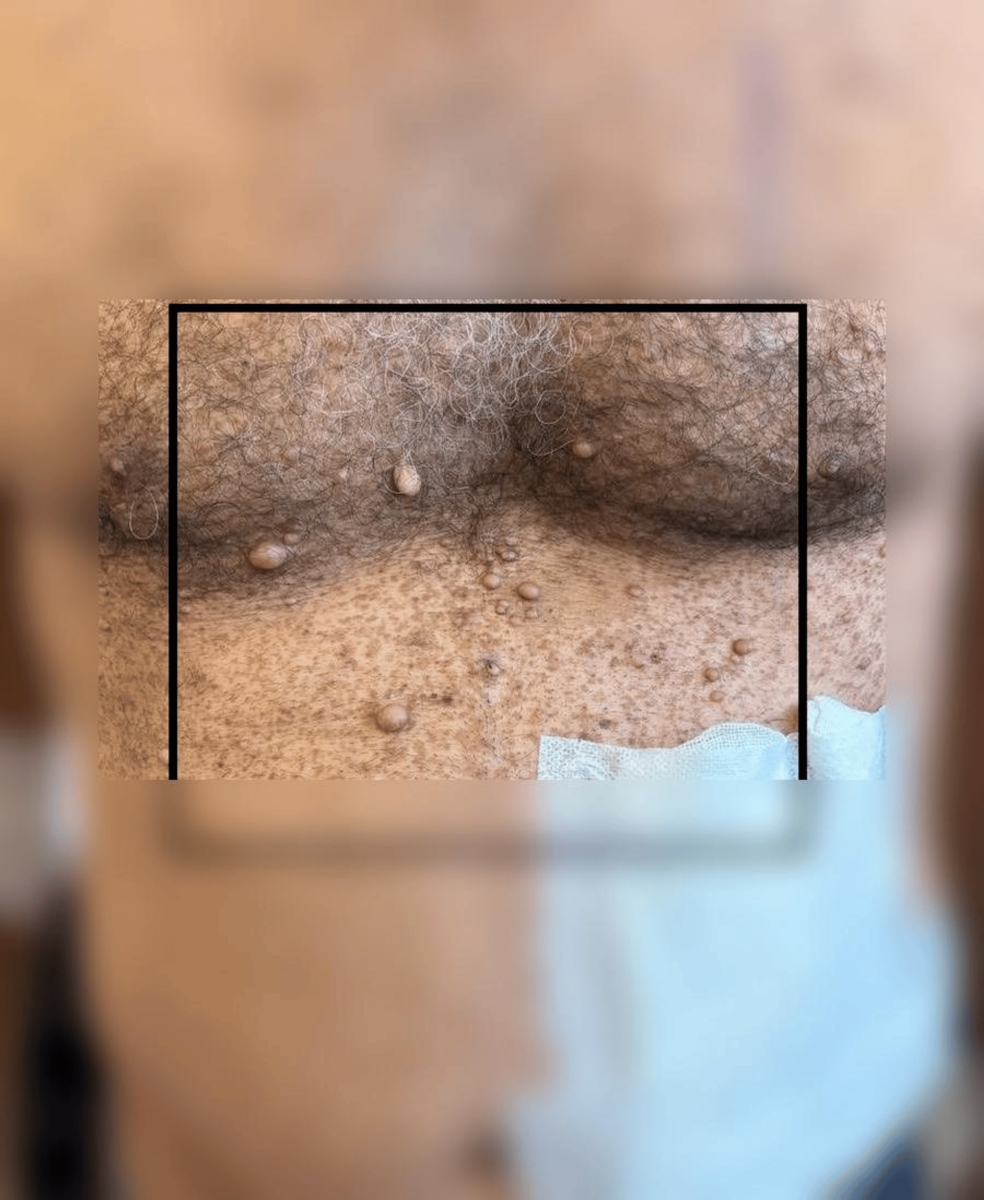
Cutaneous neurofibromas, typical dermatologic findings contributing to the clinical diagnosis of NF1

On physical examination, blood pressure measurements were persistently low, averaging approximately 90/50 mmHg on repeated assessments, while heart rate remained within normal limits. Dermatologic examination revealed multiple café-au-lait macules, axillary freckling, and numerous cutaneous neurofibromas. In addition, a giant plexiform neurofibroma measuring approximately 10 cm was observed on the right hip. Ophthalmologic examination demonstrated bilateral Lisch nodules. These findings fulfilled the National Institutes of Health diagnostic criteria for NF1 [1,3]. Histopathological examination of a sacral skin lesion confirmed neurofibroma, further supporting the clinical diagnosis. The characteristic cutaneous and ophthalmologic findings are shown in figures (Figures 1-3).

Ten years prior, the patient was diagnosed with type 2 diabetes mellitus. At presentation, he was referred for metabolic evaluation, during which persistent hypotension was noted. Subsequent diagnostic workup included biochemical testing and adrenal imaging. Following this evaluation, the patient underwent left adrenalectomy. Histopathological examination confirmed pheochromocytoma, and biochemical parameters normalized postoperatively.

Diagnostic assessment

Given the presence of persistent unexplained hypotension in a patient with clinically evident NF1, an underlying endocrine etiology was considered. Although the patient lacked classical symptoms of catecholamine excess, the known association between NF1 and pheochromocytoma prompted biochemical screening. Endocrine evaluation revealed adequate suppression of serum cortisol following a 1 mg overnight dexamethasone suppression test. Plasma renin activity was 0.38 ng/mL/h, and aldosterone concentration was 4 ng/dL. Catecholamine analysis demonstrated isolated elevation of plasma metanephrine (393.8 ng/L; reference range 0-90 ng/L). In contrast, plasma normetanephrine, epinephrine, norepinephrine, dopamine, and other catecholamine metabolites were within normal limits. The 24-hour urinary analysis confirmed elevated metanephrine excretion (1698 µg/day; reference range 44-261 µg/day), while other urinary catecholamines remained normal (Table 1).

**Table 1:** Laboratory findings DST: Dexamethasone Suppression Test.

Test	Value	Reference range
Plasma metanephrine	393.8 ng/L	0–90 ng/L
Plasma normetanephrine	150 ng/L	0–190 ng/L
Plasma epinephrine	27.9 ng/L	0-100 ng/L
Plasma norepinephrine	559 ng/L	0-600 ng/L
24-hour urinary metanephrine	1698 µg/day	44–261 µg/day
24-hour urinary normetanephrine	225 µg/day	44-261 µg/day
24-hour urinary epinephrine	18.7 µg/day	0-21 µg/day
24-hour urinary norepinephrine	61.2 µg/day	15-80 µg/day
Plasma renin activity	0.38 ng/mL/h	0.2–3.3 ng/mL/h
Aldosterone	4 ng/dL	4–31 ng/dL
Cortisol (after 1 mg DST)	Suppressed 1,2 µg/dL	<1.8 µg/dL

Contrast-enhanced abdominal computed tomography revealed a 3.7 × 3.7 × 4.0 cm mass in the left adrenal gland with central hypodense areas. Although a dedicated adrenal protocol with formal Hounsfield unit (HU) quantification was not available, the lesion appeared hypervascular and lacked radiologic features suggestive of a lipid-rich adenoma.

Functional imaging with Gallium-68 DOTA-octreotate Positron Emission Tomography/Computed Tomography (Ga-68 DOTATATE PET/CT) demonstrated intense somatostatin receptor uptake, supporting the diagnosis of pheochromocytoma. Histopathological examination confirmed pheochromocytoma with classical nested (“zellballen”) architecture. Tumor cells showed strong chromogranin A and synaptophysin positivity, along with S100 positivity supporting sustentacular cell distribution. The Ki-67 proliferation index was low (approximately 0-1%). The tumor measured 4.7 × 3.3 × 3.2 cm. Capsular invasion was present (1+), and periadrenal adipose tissue invasion was noted (2+), while lymphovascular invasion was not identified. The surgical margin was close (approximately 250 µm). The Pheochromocytoma of the Adrenal gland Scaled Score (PASS) was calculated as six, and the Grading of Adrenal Pheochromocytoma and Paraganglioma (GAPP) score was three, indicating moderately differentiated tumor features.

Diagnosis and prognosis

Based on imaging findings, biochemical results, and histopathological confirmation, a diagnosis of pheochromocytoma was established. The tumor was localized, and the surgical prognosis was considered favorable.

Therapeutic intervention

The patient underwent left adrenalectomy following standard preoperative preparation. Pharmacologic alpha-adrenergic blockade was carefully titrated due to baseline hypotension. The surgical procedure was completed without complications.

Follow-up and outcomes

Postoperatively, plasma metanephrine levels normalized, and blood pressure remained stable without worsening hypotension. The patient reported subjective improvement in overall well-being. No adverse or unanticipated events were observed during follow-up.

Genetic analysis

Genetic testing was performed using next-generation sequencing (NGS) technology (Illumina MiSeq platform, Illumina, Inc., San Diego, California, US) targeting the NF1 gene (NM_001042492.3). All coding exons and exon-intron boundary regions were analyzed from peripheral blood DNA. A heterozygous frameshift variant, c.3322dup p.(Thr1108AsnfsTer9), was identified. This duplication introduces a premature termination codon and is predicted to result in truncation of the neurofibromin protein. The variant was classified as “likely pathogenic” according to American College of Medical Genetics and Genomics (ACMG) / Association for Molecular Pathology (AMP) criteria. It has not been previously reported in the literature or major population databases, supporting its classification as a novel variant. Genetic analysis was performed in an accredited laboratory participating in European Molecular Genetics Quality Network (EMQN) and Genomics Quality Assessment (GenQA) quality assurance programs.

## Discussion

This case illustrates three clinically relevant aspects: (1) persistent hypotension in pheochromocytoma, (2) isolated metanephrine elevation, and (3) a novel truncating NF1 mutation. Although hypertension is the hallmark of pheochromocytoma, normotensive presentations occur in up to 10-15% of cases, and rare hypotensive cases have been described [6,7,9]. Proposed mechanisms include epinephrine-predominant secretion leading to β2-mediated vasodilation, chronic catecholamine-induced adrenergic receptor downregulation, or intravascular volume depletion [11]. In our patient, isolated metanephrine elevation suggests epinephrine-dominant tumor secretion, potentially explaining persistent hypotension. Previous reports have described normotensive pheochromocytoma in NF1; however, persistent hypotension remains exceptionally rare. Similar atypical presentations have been attributed to epinephrine-predominant secretion or altered adrenergic receptor sensitivity, although data remain limited.

Biochemically, plasma free metanephrines have high sensitivity for pheochromocytoma detection [6]. Isolated elevation of metanephrine with normal normetanephrine is uncommon but may occur in adrenal tumors with selective catecholamine metabolism patterns. This underscores the importance of interpreting metanephrine results independently rather than requiring global catecholamine elevation. These mechanisms remain largely hypothesis-based and are supported by indirect clinical observations rather than direct functional evidence.

Genetically, truncating NF1 mutations have been associated with increased tumor burden and more severe phenotypes [14]. The identified variant was absent from major population databases such as ClinVar by National Center for Biotechnology Information and The Human Gene Mutation Database (HGMD) [17,18] supporting its novelty. Reporting novel pathogenic variants contributes to refinement of genotype-phenotype correlations and improves future diagnostic interpretation [13,15]. Variant classification was performed according to established ACMG/AMP standards [16]. These standardized frameworks are essential for consistent interpretation of sequence variants and are widely used in clinical genetics practice [16]. NF1-associated tumors require structured surveillance strategies due to their heterogeneous clinical behavior and lifelong tumor risk [19]. Further accumulation of such cases may help clarify genotype-phenotype correlations in NF1-associated tumors.

## Conclusions

This case highlights that pheochromocytoma in patients with NF1 may present with persistent hypotension and isolated biochemical abnormalities, representing an atypical and potentially misleading clinical scenario. Such presentations may delay diagnosis, particularly in the absence of classic catecholamine-related symptoms. Our findings emphasize the importance of maintaining a high index of suspicion for pheochromocytoma in NF1 patients, even when hypertension is not present, especially in the setting of unexplained hemodynamic alterations. Recognition of these atypical patterns may facilitate earlier diagnosis and appropriate management.

However, as this report describes a single case, the observations should be interpreted with caution and primarily serve to raise clinical awareness rather than establish definitive diagnostic or therapeutic recommendations. Further studies and accumulation of similar cases are needed to better characterize the clinical spectrum, underlying mechanisms, and optimal evaluation strategies of atypical pheochromocytoma in NF1. In addition, the identification of a novel pathogenic NF1 frameshift mutation contributes to the expanding mutational spectrum of the disease and may provide further insight into genotype-phenotype correlations in NF1-associated tumors.
